# Comparison of chemotherapy and chidamide combined with chemotherapy in patients with untreated angioimmunoblastic T-cell lymphoma

**DOI:** 10.3389/fonc.2024.1373127

**Published:** 2024-04-09

**Authors:** Simeng Gu, Xin Wang, Jingqiu Zhou, Shanshan Du, Ting Niu

**Affiliations:** ^1^ Department of Hematology, West China Hospital, Sichuan University, Chengdu, China; ^2^ Department of Hematology, Chengdu Seventh People’s Hospital, Chengdu, China

**Keywords:** angioimmunoblastic T-cell lymphoma, chidamide, chemotherapy, frontline treatment, ASCT

## Abstract

**Background:**

Angioimmunoblastic T-cell lymphoma (AITL) is characterized by high recurrence rates and poor prognosis, and effective first-line treatment is lacking. Recently, histone deacetylase inhibitors (HDACi), such as chidamide, have been found to induce durable remissions in AITL patients.

**Methods:**

Patients with untreated AITL from March 2015 to March 2023 were retrospectively collected and divided into chemotherapy (ChT) group and chidamide combined with chemotherapy (C-ChT) group based on the first-line treatment received. The comparison of efficacy and safety between the two groups was conducted.

**Results:**

86 patients with newly diagnosed AITL were enrolled, in which 35 patients were in the ChT group and 51 in the C-ChT group. The objective response rate (ORR) of C-ChT group was significantly higher than that of ChT group (84.3% vs. 60%, *P*= 0.011), and had superior progression-free survival (PFS) (27 months vs. 12 months, *P*= 0.025). However, no significant difference in overall survival (OS) was observed between the two groups (*P*= 0.225). In addition, the responding patients who received autologous stem cell transplantation (ASCT) had superior PFS compared to those who did not (*P*= 0.015).

**Conclusions:**

Compared with ChT regimen, C-ChT regimen was well tolerated and had superior ORR and PFS in patients with untreated AITL. ASCT may contribute to longer PFS in remission patients.

## 1 Introduction

Angioimmunoblastic T-cell lymphoma (AITL), a lymphoma characterized by the T follicular helper (TFH) phenotype (1), is the second most common peripheral T-cell lymphomas (PTCLs), comprising approximately 1-2% of non-Hodgkin’s lymphomas (NHLs) and 15-20% of PTCLs worldwide (2, 3). AITL is associated with a poor prognosis, with a 5-year overall survival (OS) rate of only 30%. AITL exhibits notable geographic variability; in the Chinese population, AITL accounts for approximately 1.6% of NHLs and 37.4% of PTCLs, with a 5-year OS rate of about 34.9% (4, 5). Anthracycline-based chemotherapy, such as cyclophosphamide, doxorubicin, vincristine, and prednisone (CHOP) or CHOP-like regimens, is mostly used for first-line treatment of AITL. Unlike the good efficacy achieved in B lymphomas, AITL shows a poor response to traditional chemotherapy (ChT), requiring urgent exploration of novel drugs and different drug combination regimens (3).

Epigenetic dysregulation has been found to play an important role in the pathogenesis of PTCL, especially AITL (6, 7). Preclinical and clinical trial data suggest that histone deacetylase inhibitors (HDACi), as an epigenetic agent, result in more durable and stable remissions in the treatment of AITL, compared to other subtypes of PTCL (8, 9). Chidamide, a novel oral selective histone deacetylase inhibitor, promotes tumor cell growth arrest and apoptosis, which exhibits antitumor activity in relapsed or refractory PTCL (10, 11). A pivotal phase II trial demonstrated an overall remission rate (ORR) of 28% for chidamide monotherapy in relapsed or refractory PTCL (r/r PTCL), with AITL demonstrating a higher ORR of 50% (12). Based on the results of this trial, the China Food and Drug Administration approved chidamide in December 2014 for patients with r/r PTCL. A Chinese multicenter real-world study including 383 patients with r/r PTCL showed that the ORR and disease control rate (DCR) were 39.06% and 64.45% for chidamide monotherapy, and 51.18% and 74.02% for combination chemotherapy, respectively (13). It is noteworthy that chidamide has some toxicities. Common adverse events (AEs) related to chidamide include hematological abnormalities, fatigue, nausea/vomiting, and abnormal hepatic function. Transient prolongations of the QTc interval have been occasionally observed in patients (12, 13). Most AEs are grade 1-2 and can be managed with supportive therapy and suspension of chidamide.

Attempts to use chidamide for the first-line treatment of PTCL are also underway (14–16). A prospective phase Ib/II study showed that chidamide, in combination with the CHOEP regimen, for the first-line treatment of PTCL, demonstrated an ORR of 60.2%, a complete response (CR) rate of 40.7%, and 1-year and 3-year PFS rates of 49.9% and 32.8%, respectively (15). However, clinical data for first-line treatment of AITL with chidamide are lacking due to the low prevalence of AITL. In the present study, we compared the efficacy and toxicity of conventional chemotherapy (ChT) and chidamide combined with chemotherapy (C-ChT) in the frontline treatment of AITL. The role of autologous stem cell transplantation (ASCT) in first-line treatment and the prognostic factors of patients with newly diagnosed AITL were also assessed.

## 2 Methods

### 2.1 Patients

**Figure 1:**
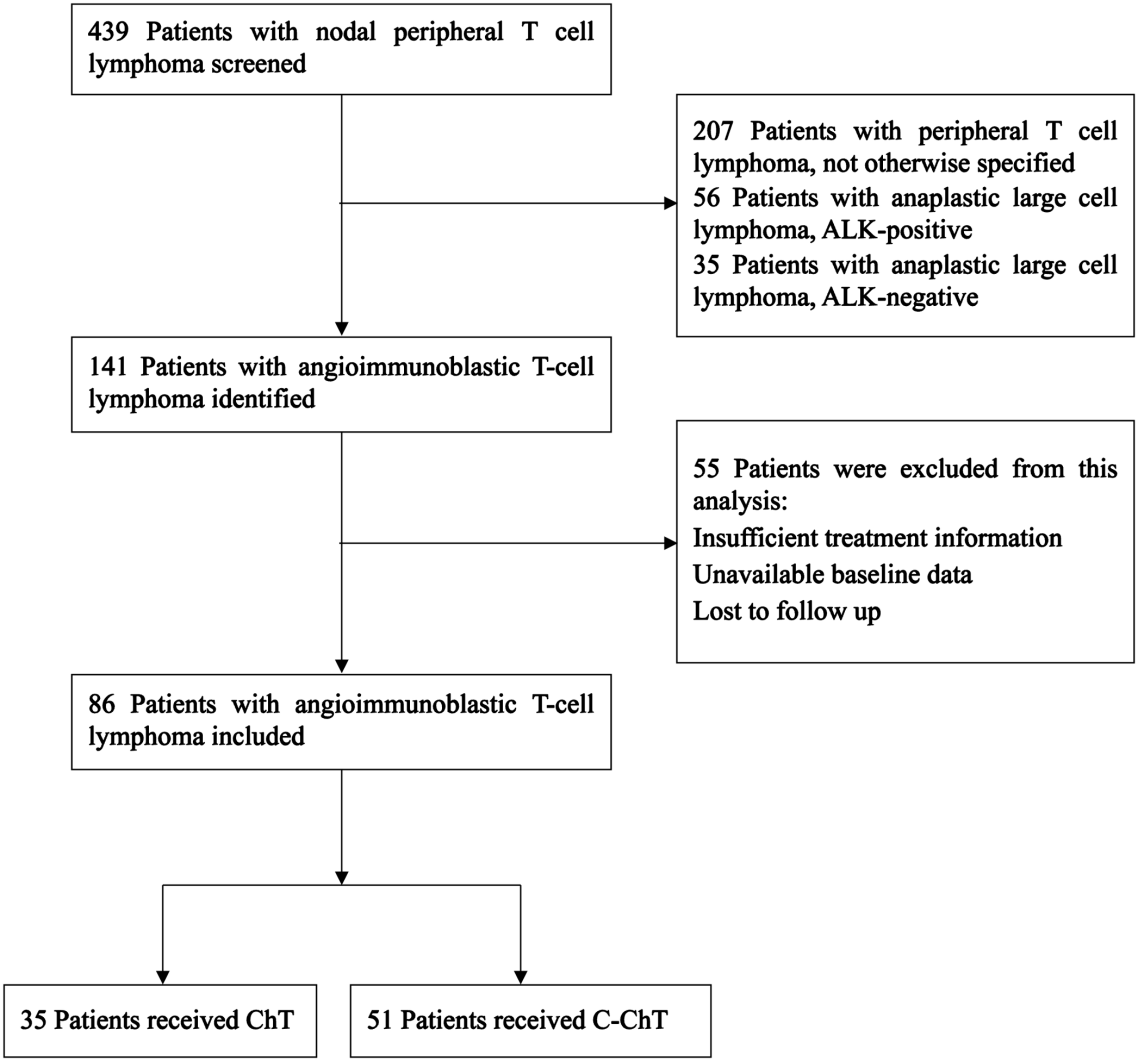
The flow chart of patient inclusion.

Our study aimed to retrospectively analyze patients newly diagnosed with AITL and receiving first-line treatment at West China Hospital of Sichuan University between March 2015 and March 2023. Ultimately, 439 patients with nodal PTCL were screened, of which 86 patients with newly diagnosed AITL were retrospectively analyzed, as shown in **Figure 1**. The inclusion criteria were as follows: (1) clear pathologic diagnosis of AITL; (2) completion of at least 3 or more chemotherapy treatments; (3) complete examination test and staging information. The exclusion criteria were: unavailability of treatment or baseline information; loss to follow-up. This study was approved by the institutional ethical review board of West China Hospital of Sichuan University and was exempted from the informed consent requirement because anonymized data were used.

### 2.2 Study design

Our study retrospectively compared the efficacy and safety of chemotherapy regimens with or without chidamide in patients with untreated AITL. Patients were divided into chemotherapy (ChT) group and chidamide combined with chemotherapy (C-ChT) group according to the first-line chemotherapy regimens they received. Their baseline information, treatment information, adverse effects and prognostic factors were retrospectively collected and analyzed. Moreover, in patients who achieved complete remission (CR) or partial remission (PR) after frontline treatment, survival comparisons were performed based on whether or not they received ASCT.

The clinical data collected included age, gender, B symptoms, Ann Arbor stage, Eastern Cooperative Oncology Group (ECOG) performance status, the International Prognostic Index (IPI), the Prognostic Index for PTCL-U patients (PIT), modified-PIT score, bone marrow invasion state, whether or not combined with hemophagocytic syndrome, extranodal invasion, serum lactate dehydrogenase (LDH) level, albumin level, complete blood count, treatment regimen, treatment response, and survival status.

The primary endpoint was progression-free survival (PFS), and the secondary endpoints were overall survival (OS), ORR and CR rate. PFS was defined as the time interval from the date of treatment initiation to the first observation of disease progression or death from any cause. Progression was defined as relapse from CR if the patient had achieved CR, or as progressive disease if the patient had not achieved CR. OS was defined as the time from diagnosis to death from any cause or the last follow-up. Efficacy evaluations were performed every two to three cycles by computed tomography, magnetic resonance imaging and positron emission computed tomography, and were evaluated as CR, PR, stable disease (SD), or progressive disease (PD) according to the 2014 Lugano Classification lymphoma response criteria. The toxicity grading was conducted according to the Common Terminology Criteria for Adverse Events version 4.0.

### 2.3 Statistical analysis

The differences between categorical variables were analyzed by the χ2 test or Fisher’s exact test. PFS and OS analyses were performed using the Kaplan-Meier method, and comparisons of survival between groups were performed using the log-rank test. Cox regression analyses were used for the identification of prognostic factors, with univariate Cox regression analyses firstly performed to identify the significant variables, and then significant variables were further identified in multivariate Cox analyses to determine independent prognostic factors.

All analyses were performed using IBM SPSS Statistics 26.0 software. A *P*-value <0.05 was considered statistically significant throughout the analysis.

## 3 Results

### 3.1 Baseline characteristics

86 patients with newly diagnosed AITL were included in the present study, with 35 and 51 patients in the ChT group and the C-ChT group, respectively (**Table 1**). The baseline characteristics of the patients were shown in **Table 1**. The median age of all patients was 59 years (range 41-84 years), with a median age of 61 years (range 43-78 years) in the ChT group and 57 years (range 41-84 years) in the C-ChT group. Of the 86 patients, 59 (68.6%) were males, with 27 (77.1%) and 32 (62.7%) in ChT and C-ChT groups, respectively. About half of the patients showed B symptoms at the time of diagnosis. 89.5% of patients had advanced stage disease, with 82.9% and 94.1% in the ChT and C-ChT groups, respectively. Bone marrow invasion was observed in 39.5% of patients (34.3% in the ChT group and 43.1% in the C-ChT group), and 70.9% of patients had extranodal involvement (68.6% and 72.5% in the ChT and C-ChT groups, respectively).

**Table 1:** The baseline characteristics of 86 AITL patients.

	Overall (n=86)	ChT (n=35)	C+ChT (n=51)	P
Age				
Median (range)	59 (41-84)	61 (43-78)	57 (41-84)	P=0.208
≥60	41 (47.7%)	19 (54.3%)	22 (43.1%)	P=0.309
Gender				P=0.158
Male	59 (68.6%)	27 (77.1%)	32 (62.7%)	
Female	27 (31.4%)	8 (22.9%)	19 (37.3%)	
Time of treatment				P=0.078
March 2015 - March 2019	21 (24.4%)	12 (34.3%)	9 (17.6%)	
April 2019 - March 2023	65 (75.6%)	23 (65.7%)	42 (82.4%)	
B symptoms				P=0.826
Yes	43 (50.0%)	18 (51.4%)	25 (49.0%)	
No	43 (50.0%)	17 (48.6%)	26 (51.0%)	
Ann Arbor stage				P=0.188
I/II	9 (10.5%)	6 (17.1%)	3 (5.9%)	
III/IV	77 (89.5%)	29 (82.9%)	48 (94.1%)	
ECOG score				P=0.416
0-1	63 (73.3%)	24 (68.6%)	39 (76.5%)	
≥2	23 (26.7%)	11 (31.4%)	12 (23.5%)	
IPI score				P=0.942
0-2	34 (39.5%)	14 (40.0%)	20 (39.2%)	
3-5	52 (60.5%)	21 (60.0%)	31 (60.8%)	
PIT score				P=0.772
0-1	36 (41.9%)	14 (40.0%)	22 (43.1%)	
2-4	50 (58.1%)	21 (60.0%)	29 (56.9%)	
Modified-PIT score				P=0.676
0-1	49 (57.0%)	19 (54.3%)	30 (58.8%)	
2-4	37 (43.0%)	16 (45.7%)	21 (41.2%)	
Bone marrow involvement				P=0.409
Yes	34 (39.5%)	12 (34.3%)	22 (43.1%)	
No	52 (60.5%)	23 (65.7%)	29 (56.9%)	
Hemophagocytic syndrome				P=0.601
Yes	7 (8.1%)	4 (11.4%)	3 (5.9%)	
No	79 (91.9%)	31 (88.6%)	48 (94.1%)	
Extranodal involvement				P=0.690
Yes	61 (70.9%)	24 (68.6%)	37 (72.5%)	
No	25 (29.1%)	11 (31.4%)	14 (27.5%)	
LDH				P=0.890
Normal	41 (47.7%)	17 (48.6%)	24 (47.1%)	
Elevated	45 (52.3%)	18 (51.4%)	27 (52.9%)	
Albumin				P=0.275
≥35	62 (72.1%)	23 (65.7%)	39 (76.5%)	
<35	24 (27.9%)	12 (34.3%)	12 (23.5%)	
Hemoglobin				P=0.639
Normal	37 (43.0%)	14 (40.0%)	23 (45.1%)	
Decreased	49 (57.0%)	21 (60.0%)	28 (54.9%)	
Platelet				P=0.678
Normal	72 (83.7%)	30 (85.7%)	42 (82.4%)	
Decreased	14 (16.3%)	5 (14.3%)	9 (17.6%)	

No significant differences were observed between the ChT group and the C-ChT group in terms of age, gender, time of treatment, B symptoms, Ann Arbor stage, PS score, IPI score, PIT score, bone marrow invasion, extranodal involvement, LDH level, albumin level, complete blood count and other baseline characteristics, and the two groups were well balanced and comparable.

24 (68.6%) and 41 (80.4%) in the ChT and C-ChT groups, respectively, received first-line CHOP regimen chemotherapy, which included cyclophosphamide, doxorubicin, vincristine, and prednisone. 5 (14.3%) in the ChT group received the GCHOP regimen (gemcitabine+CHOP), and 6 (17.1%) received the ECHOP regimen chemotherapy (etoposide+CHOP), and 3 (8.6%) patients received ASCT consolidation therapy in first remission. Meanwhile, 5 (9.8%) patients in the C-ChT group received the GCHOP chemotherapy, 5 (9.8%) patients received the ECHOP regimens, and 9 (17.6%) patients received ASCT in first remission.

### 3.2 Response

**Figure 2:**
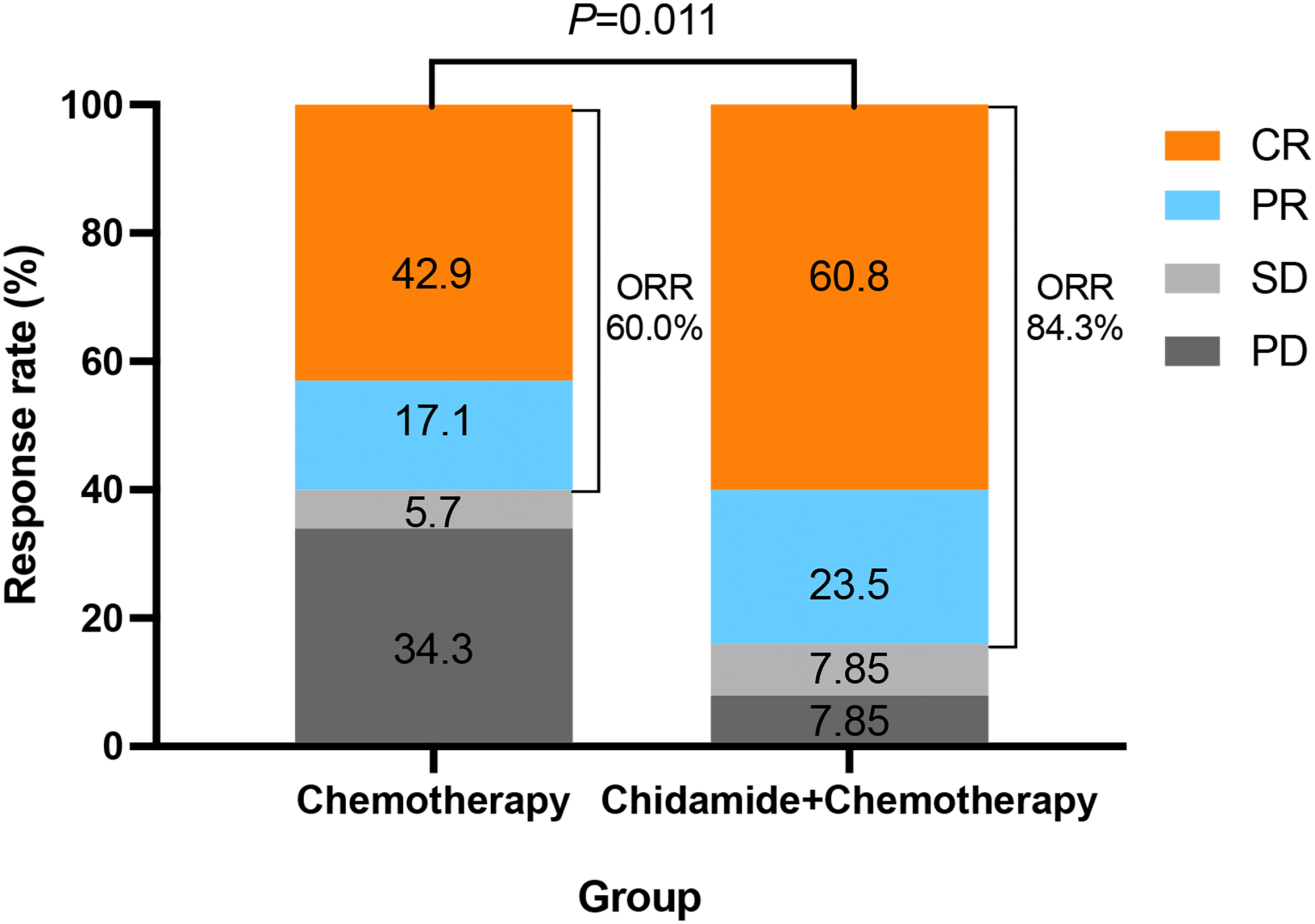
Therapeutic response in ChT and C-ChT groups.

The ORR of all patients was 74.4%, with the CR rate of 53.5%. The ORR of the ChT group and the C-ChT group were 60.0% and 84.3%, respectively (*P*=0.011, **Figure 2**), and the CR rates were 42.9% and 60.8%, respectively (*P*=0.102). Compared with the ChT group, the C-ChT regimen could significantly improve the ORR of patients with untreated AITL, although no significant difference in the CR rate was observed.

25 (39.1%) of the 64 patients who achieved CR/PR eventually relapsed, and the median duration of response (CR+PR) has not been reached. 9 (42.9%) of the 21 patients who reached objective remission in the ChT group faced relapse, and all of them relapsed within 1 year. In the ChT group, 5 of the 9 relapsed patients received second-line treatment with chidamide-containing chemotherapy. 2 patients achieved objective remission, 1 of which was CR and planned to undergo ASCT as consolidation therapy, and the other was PR and is currently in maintenance therapy with chidamide. 1 patient was SD and progressed after a duration of 16 months, while another patient died due to PD. 1 patient discontinued chidamide due to grade 3 pulmonary infection. 16 (37.2%) of the 43 patients in the C-ChT group who reached CR/PR eventually relapsed, with the longest duration of remission being >30 months. No significant difference was observed between the two groups in relapse rate (*P*=0.664).

### 3.3 Survival

**Figure 3:**
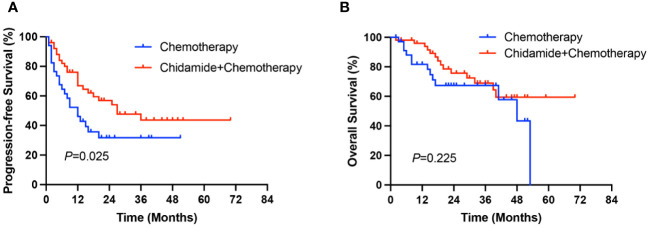
Comparison of PFS **(A)** and OS **(B)** between the ChT and Chidamide+ChT groups.

After a median follow-up of 33 months (range 2-70 months), the median PFS and OS of all patients were 18 months and 53 months, respectively. The median PFS in the ChT group was 12 months (range 0-51 months), and the 1-year and 3-year PFS rates were 44.8% and 30.8%, respectively. The median PFS in the C-ChT group was 27 months (range 1-70 months), and the 1-year and 3-year PFS rates were 66.8% and 43.7%, respectively. There was significant difference between the two groups (12 vs 27 months, *P*=0.025, **Figure 3A**), with the C-ChT group having significantly longer PFS in AITL patients.

The 1-year and 3-year OS rates for patients in the ChT group were 78.3% and 57.8%, respectively, with a median OS of 48 months (range 2-53 months), while the 1-year and 3-year OS rates in the C-ChT group were 93.8% and 64.4%, respectively, with a median OS of not reached. However, no significant difference in OS was observed between the two groups (48 months vs not reached, *P*=0.225, **Figure 3B**).

3 patients (8.6%) in the ChT group and 9 patients (17.6%) in the C-ChT group underwent ASCT as part of their subsequent treatment, and there was no significant difference between the two groups regarding ASCT treatment (*P*=0.381).

### 3.4 Adverse events

Hematological toxicities were the most common adverse events (AEs) observed. In the ChT and C-ChT group, the incidence of grade 3/4 thrombocytopenia was 20% and 29.4% (*P*=0.326), the incidence of grade 3/4 anemia was 20% and 27.5% (*P*=0.429), and the incidence of grade 3/4 neutropenia was 40%, and 47.1% (*P*=0.517), respectively. No significant differences were observed between the two groups (**Table 2**). The incidence of grade 3/4 infections was not high, with 17.1% and 23.5% in the ChT and C-ChT groups (*P*=0.474, **Table 2**), respectively. Toxic reactions such as elevated creatinine, fatigue, nausea and vomiting, and diarrhea occurred in some patients, all of which were grade 1-2 and recovered with supportive therapy. Cardiac toxicity was not observed in any patient. No significant differences in the frequency of AEs were observed between the two groups (all *P*>0.05). There were no reports of treatment-related deaths.

**Table 2:** Adverse events in the ChT and C-ChT groups.

	Overall (n=86)	ChT (n=35)	C-ChT (n=51)	P
Thrombocytopenia				0.326
≥ Grade 3	22 (25.6%)	7 (20.0%)	15 (29.4%)	
Anemia				0.429
≥ Grade 3	21 (24.4%)	7 (20.0%)	14 (27.5%)	
Neutropenia				0.517
≥ Grade 3	38 (44.2%)	14 (40.0%)	24 (47.1%)	
Infections				0.474
≥ Grade 3	18 (20.9%)	6 (17.1%)	12 (23.5%)	
Elevated ALT/AST				0.407
≥ Grade 3	1 (1.2%)	1 (2.9%)	0 (0)	
Elevated Creatinine				0.185
Grade 1-2	8 (9.3%)	1 (2.9%)	7 (13.7%)	
Fatigue				0.780
Grade 1-2	21 (24.4%)	8 (22.9%)	13 (25.5%)	
Nausea/vomiting				0.387
Grade 1-2	24 (27.9%)	8 (22.9%)	16 (31.4%)	
Diarrhea				0.960
Grade 1-2	6 (7.0%)	3 (8.6%)	3 (5.9%)	

### 3.5 Prognostic factor analysis

Univariate Cox analysis of PFS suggested that first-line chemotherapy regimen, B symptoms and platelet level may be prognostic factors (*P*=0.030, 0.043, 0.019, respectively, **Table 3**). Multivariate analysis showed that first-line chemotherapy regimen and platelet level were independent prognostic factors of PFS in patients with untreated AITL. The patients treated with chidamide combined with chemotherapy had superior PFS (HR 0.554, 95%CI 0.311-0.987, *P*=0.045, **Table 3**) compared with conventional chemotherapy. At the time of diagnosis, the risk of progression in patients with low platelet levels was 2.083 times higher than in patients with normal platelet levels (HR 2.083, 95% CI 1.042-4.163, *P*=0.038, **Table 3**).

**Table 3:** Univariate and multivariate Cox analyses of PFS in patients with newly diagnosed AITL.

Variable	Univariate analysis		Multivariate analysis	
	HR (95% CI)	P-value	HR (95% CI)	P-value
Age (≥60 vs <60 years)	1.271 (0.716-2.257)	0.413		
Treatment (C+ChT vs ChT)	0.528 (0.297-0.940)	0.030	0.554 (0.311-0.987)	0.045
Chemotherapy backbone (CHOP vs non-CHOP)	0.842 (0.444-1.597)	0.599		
Gender (female vs male)	0.676 (0.344-1.328)	0.255		
B symptoms (yes vs no)	1.840 (1.020-3.318)	0.043	1.658 (0.912-3.014)	0.098
Ann Arbor stage (III/IV vs I/II)	1.896 (0.588-6.111)	0.284		
ECOG score (≥2 vs 0-1)	1.514 (0.827-2.771)	0.178		
IPI score (3-5 vs 0-2)	1.632 (0.882-3.020)	0.119		
PIT score (2-4 vs 0-1)	1.253 (0.695-2.260)	0.453		
Modified-PIT score (2-4 vs 0-1)	1.420 (0.800-2.520)	0.231		
Bone marrow involvement (yes vs no)	1.344 (0.753-2.401)	0.317		
Hemophagocytic syndrome (yes vs no)	2.274 (0.961-5.382)	0.062		
Extranodal involvement (yes vs no)	1.576 (0.801-3.101)	0.187		
LDH (elevated vs normal)	1.325 (0.743-2.363)	0.341		
Albumin (<35 vs ≥35)	1.351 (0.730-2.498)	0.338		
Hemoglobin (decreased vs normal)	1.140 (0.632-2.055)	0.663		
Platelet (decreased vs normal)	2.281 (1.148-4.530)	0.019	2.083 (1.042-4.163)	0.038

Patients with B symptoms, bone marrow invasion, albumin <35 and reduced platelet levels at the time of diagnosis tended to have inferior OS in the univariate analysis (*P*=0.011, 0.025, 0.033 and 0.048, respectively, **Table 4**), however, further multivariate analysis failed to identify independent prognostic factors for OS.

**Table 4:** Univariate and multivariate Cox analyses of OS in patients with newly diagnosed AITL.

Variable	Univariate analysis		Multivariate analysis	
	HR (95% CI)	P-value	HR (95% CI)	P-value
Age (≥60 vs <60 years)	1.516 (0.699-3.284)	0.292		
Treatment (C+ChT vs ChT)	0.630 (0.296-1.342)	0.231		
Chemotherapy backbone (CHOP vs non-CHOP)	0.665 (0.303-1.458)	0.308		
Gender (female vs male)	1.199 (0.523-2.747)	0.668		
B symptoms (yes vs no)	3.068 (1.293-7.280)	0.011	2.320 (0.928-5.803)	0.072
Ann Arbor stage (III/IV vs I/II)	23.855 (0.096-5912.372)	0.259		
ECOG score (≥2 vs 0-1)	1.541 (0.697-3.407)	0.285		
IPI score (3-5 vs 0-2)	2.006 (0.843-4.774)	0.116		
PIT score (2-4 vs 0-1)	1.362 (0.621-2.988)	0.440		
Modified-PIT score (2-4 vs 0-1)	1.370 (0.634-2.958)	0.423		
Bone marrow involvement (yes vs no)	2.391 (1.117-5.120)	0.025	1.699 (0.746-3.869)	0.207
Hemophagocytic syndrome (yes vs no)	2.578 (0.947-7.016)	0.064		
Extranodal involvement (yes vs no)	1.523 (0.608-3.812)	0.369		
LDH (elevated vs normal)	1.285 (0.600-2.751)	0.519		
Albumin (<35 vs ≥35)	2.325 (1.071-5.045)	0.033	1.332 (0.550-3.227)	0.526
Hemoglobin (decreased vs normal)	1.187 (0.540-2.610)	0.670		
Platelet (decreased vs normal)	2.423 (1.009-5.816)	0.048	1.374 (0.511-3.690)	0.529

### 3.6 ASCT consolidation treatment

**Figure 4:**
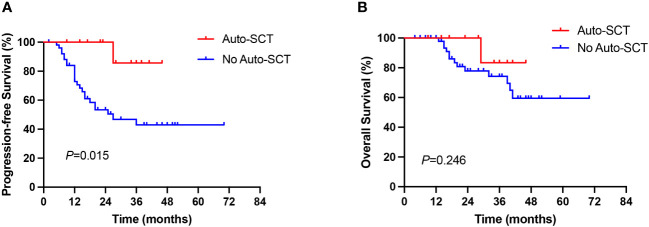
Comparison of PFS **(A)** and OS **(B)** between the ASCT and No ASCT groups.

The benefit of ASCT consolidation treatment after first remission was further evaluated. 64 of 86 newly diagnosed AITL patients achieved CR/PR, of whom 12 patients received ASCT consolidation after first remission. Patients who received ASCT consolidation in first remission significantly prolonged PFS in AITL patients compared with those who did not receive ASCT (27 months vs not reached, *P*=0.015, **Figure 4A**). However, no significant difference in OS was observed between the two groups (*P*=0.246, **Figure 4B**), which may be related to the small number of patients who underwent ASCT.

## 4 Discussion

The incidence of AITL exhibits geographical variability, being more prevalent in Western countries compared to in Asia. AITL ranks as the second most common PTCL, accounting for approximately 15-20% of PTCLs. It is defined as a lymphoma with a TFH phenotype according to the latest 2016 WHO classification of lymphomas (1, 7, 17). The pathogenesis of AITL has been found to be multistage and multistep, often combined with autoimmune disorders and epigenetic dysregulation (18, 19). Traditional anthracycline-based conventional chemotherapy, like CHOP or CHOP-like regimens, has performed disappointingly in the frontline treatment of AITL. Recently, immunomodulators, epigenetic drugs, and targeted therapies have shown great potential in AITL (20, 21), especially HDAC inhibitors such as chidamide and romidepsin, which have been approved in patients with relapsed or refractory PTCLs (12, 22).

An international phase III clinical trial comparing romidepsin plus CHOP vs CHOP for the first-line treatment of PTCL showed that HDACi combined with chemotherapy failed to significantly improve survival (23). However, exploratory analysis of the study data revealed that HDACi has the potential to achieve a more durable and deeper response in AITL. Several other studies have confirmed this finding, suggesting that lymphomas exhibiting a TFH phenotype benefit more from HDACi application (8, 9, 24), which may be explained by the fact that the pathogenesis of AITL often involves epigenetic dysregulation.

Chidamide, a novel oral HDACi independently designed in China, is very promising in the treatment of AITL, however, clinical data on the first-line treatment of AITL with chidamide is still lacking. Based on this, the present study has shed light on the potential benefits of using chidamide in the combinatorial chemotherapy in patients with untreated AITL.

Previous studies have shown the potential synergistic effects of HDACi and cytotoxic drugs (25, 26). However, a subset of patients in our cohort did not receive chidamide due to financial constraints. In our study, the chidamide combined with conventional chemotherapy group had significantly superior ORR and PFS compared to the conventional chemotherapy group, with an ORR of 84.3% and a median PFS of 27 months. Due to limited follow up, this study is unable to evaluate for the OS. Furthermore, given the retrospective design and limited sample size of this study, it remains uncertain whether improvements in ORR and PFS can be attributed to the drug effects of chidamide, or if they are influenced by other confounding variables and selection bias.

The high recurrence rate of AITL is also an important factor in the poor prognosis of AITL, which has been reported to be as high as 30% (27), similar to the present study. In our study, 39.1% of patients relapsed after their first remission. The failure of chidamide combined with chemotherapy to significantly reduce the relapse rate may explain why the significant improvement in ORR rate ultimately failed to translate into prolonged OS.

AITL mostly occurs in the elderly population, with a median age of 65 years. Most patients are diagnosed at advanced stages, often combined with autoimmune anemia, elevated LDH, and bone marrow and extra-nodal invasions, leading to a dismal prognosis (2, 27). Currently, the International Prognostic Index (IPI) is mostly used to assess the prognosis of patients with AITL; however, unlike its accuracy in the prognostic assessment of B-cell lymphoma, it works poorly in AITL patients (2). Recent prognostic studies have indicated that thrombocytopenia, B-symptoms, rapid progression, bone marrow invasion, and hypoproteinemia are adverse prognostic indicators in patients with PTCL (28, 29), which is similar to the present study. Patients whose frontline regimen did not contain chidamide or who had decreased platelet count were at a higher risk of progression in our study, and in addition, patients with B symptoms, bone marrow invasion, hypoproteinemia, and thrombocytopenia may have inferior OS.

The precise therapeutic role and optimal timing of ASCT in AITL have yet to be fully determined. Several studies have shown that consolidation therapy with ASCT in PTCL patients following initial remission contributes to prolonged PFS, which is similar to our findings (30–32). However, in our study, due to the poor economic situation and insufficient understanding of ASCT, only a very small number of patients underwent ASCT (n=12).

Undeniably, there are some limitations to the present study. First, this study is a retrospective study with some inherent confounding variables, and second, the small sample size limits further analysis in depth. However, the low prevalence of AITL makes it difficult to conduct large-scale prospective randomized trials. Still, our study provides some insights into the diagnosis and treatment of patients with AITL. Large-scale randomized controlled trials for different subtypes of PTCL are necessary in the future.

## 5 Conclusions

Compared with conventional chemotherapy regimens, first-line treatment of AITL patients with chidamide in combination with chemotherapy may improve ORR and PFS without increasing chemotherapy toxicity. Moreover, ASCT may contribute to longer PFS in remission patients. It is essential to conduct a larger and randomized study in the future to further identify superior treatment strategies.

## Data availability statement

The raw data supporting the conclusions of this article will be made available by the authors, without undue reservation.

## Ethics statement

The studies involving humans were approved by the institutional ethical review board of West China Hospital of Sichuan University. The studies were conducted in accordance with the local legislation and institutional requirements. Written informed consent for participation was not required from the participants or the participants’ legal guardians/next of kin in accordance with the national legislation and institutional requirements.

## Author contributions

SG: Writing – original draft, Data curation, Formal analysis, Methodology. XW: Data curation, Writing – original draft, Formal analysis, Methodology. JZ: Data curation, Writing – original draft. SD: Data curation, Writing – original draft. TN: Funding acquisition, Writing – review & editing, Supervision.

## References

[1] Swerdlow SH Campo E Pileri SA Harris NL Stein H Siebert R. The 2016 revision of the World Health Organization classification of lymphoid neoplasms. Blood. (2016) 127:2375–90. doi: 10.1182/blood-2016-01-643569 PMC4874220 26980727

[2] Lunning MA Vose JM. Angioimmunoblastic T-cell lymphoma: the many-faced lymphoma. Blood. (2017) 129:1095–102. doi: 10.1182/blood-2016-09-692541 28115369

[3] Federico M Rudiger T Bellei M Nathwani BN Luminari S Coiffier B. Clinicopathologic characteristics of angioimmunoblastic T-cell lymphoma: analysis of the international peripheral T-cell lymphoma project. J Clin Oncol. (2013) 31:240–6. doi: 10.1200/JCO.2011.37.3647 PMC3532394 22869878

[4] Sun J Yang Q Lu Z He M Gao L Zhu M. Distribution of lymphoid neoplasms in China: analysis of 4,638 cases according to the World Health Organization classification. Am J Clin Pathol. (2012) 138:429–34. doi: 10.1309/AJCP7YLTQPUSDQ5C 22912361

[5] Liu W Ji X Song Y Wang X Zheng W Lin N. Improving survival of 3760 patients with lymphoma: Experience of an academic center over two decades. Cancer Med. (2020) 9:3765–74. doi: 10.1002/cam4.3037 PMC7286476 32281275

[6] O'Connor OA Bhagat G Ganapathi K Pedersen MB D'Amore F Radeski D. Changing the paradigms of treatment in peripheral T-cell lymphoma: from biology to clinical practice. Clin Cancer Res. (2014) 20:5240–54. doi: 10.1158/1078-0432.CCR-14-2020 25320373

[7] Chiba S Sakata-Yanagimoto M. Advances in understanding of angioimmunoblastic T-cell lymphoma. Leukemia. (2020) 34:2592–606. doi: 10.1038/s41375-020-0990-y PMC7376827 32704161

[8] Ghione P Faruque P Mehta-Shah N Seshan V Ozkaya N Bhaskar S. T follicular helper phenotype predicts response to histone deacetylase inhibitors in relapsed/refractory peripheral T-cell lymphoma. Blood Adv. (2020) 4:4640–7. doi: 10.1182/bloodadvances.2020002396 PMC7556143 33002132

[9] Pro B Horwitz SM Prince HM Foss FM Sokol L Greenwood M. Romidepsin induces durable responses in patients with relapsed or refractory angioimmunoblastic T-cell lymphoma. Hematol Oncol. (2017) 35:914–7. doi: 10.1002/hon.2320 PMC5763404 27402335

[10] Ning ZQ Li ZB Newman MJ Shan S Wang XH Pan DS. Chidamide (CS055/HBI-8000): a new histone deacetylase inhibitor of the benzamide class with antitumor activity and the ability to enhance immune cell-mediated tumor cell cytotoxicity. Cancer Chemother Pharmacol. (2012) 69:901–9. doi: 10.1007/s00280-011-1766-x 22080169

[11] Gong K Xie J Yi H Li W. CS055 (Chidamide/HBI-8000), a novel histone deacetylase inhibitor, induces G1 arrest, ROS-dependent apoptosis and differentiation in human leukaemia cells. Biochem J. (2012) 443:735–46. doi: 10.1042/BJ20111685 22339555

[12] Shi Y Dong M Hong X Zhang W Feng J Zhu J. Results from a multicenter, open-label, pivotal phase II study of chidamide in relapsed or refractory peripheral T-cell lymphoma. Ann Oncol. (2015) 26:1766–71. doi: 10.1093/annonc/mdv237 26105599

[13] Shi Y Jia B Xu W Li W Liu T Liu P. Chidamide in relapsed or refractory peripheral T cell lymphoma: a multicenter real-world study in China. J Hematol Oncol. (2017) 10:69. doi: 10.1186/s13045-017-0439-6 28298231 PMC5351273

[14] Gui L Cao J Ji D Zhang H Fan Q Zhu J. Chidamide combined with cyclophosphamide, doxorubicin, vincristine and prednisone in previously untreated patients with peripheral T-cell lymphoma. Chin J Cancer Res. (2021) 33:616–26. doi: 10.21147/j.issn.1000-9604.2021.05.08 PMC8580795 34815635

[15] Zhang W Su L Liu L Gao Y Wang Q Su H. The combination of chidamide with the CHOEP regimen in previously untreated patients with peripheral T-cell lymphoma: a prospective, multicenter, single arm, phase 1b/2 study. Cancer Biol Med. (2021) 18:841–8. doi: 10.20892/j.issn.2095-3941.2020.0413 PMC8330529 33755379

[16] Wang Y Zhang M Song W Cai Q Zhang L Sun X. Chidamide plus prednisone, etoposide, and thalidomide for untreated angioimmunoblastic T-cell lymphoma in a Chinese population: A multicenter phase II trial. Am J Hematol. (2022) 97:623–9. doi: 10.1002/ajh.26499 PMC9314976 35170082

[17] de Leval L Rickman DS Thielen C Reynies A Huang YL Delsol G. The gene expression profile of nodal peripheral T-cell lymphoma demonstrates a molecular link between angioimmunoblastic T-cell lymphoma (AITL) and follicular helper T (TFH) cells. Blood. (2007) 109:4952–63. doi: 10.1182/blood-2006-10-055145 17284527

[18] Tari G Lemonnier F Morschhauser F. Epigenetic focus on angioimmunoblastic T-cell lymphoma: pathogenesis and treatment. Curr Opin Oncol. (2021) 33:400–5. doi: 10.1097/CCO.0000000000000773 34230442

[19] Yao WQ Wu F Zhang W Chuang SS Thompson JS Chen Z. Angioimmunoblastic T-cell lymphoma contains multiple clonal T-cell populations derived from a common TET2 mutant progenitor cell. J Pathol. (2020) 250:346–57. doi: 10.1002/path.5376 PMC7064999 31859368

[20] Lemonnier F Safar V Beldi-Ferchiou A Cottereau AS Bachy E Cartron G. Integrative analysis of a phase 2 trial combining lenalidomide with CHOP in angioimmunoblastic T-cell lymphoma. Blood Adv. (2021) 5:539–48. doi: 10.1182/bloodadvances.2020003081 PMC7839364 33496747

[21] Lemonnier F Dupuis J Sujobert P Tournillhac O Cheminant M Sarkozy C. Treatment with 5-azacytidine induces a sustained response in patients with angioimmunoblastic T-cell lymphoma. Blood. (2018) 132:2305–9. doi: 10.1182/blood-2018-04-840538 30279227

[22] Coiffier B Pro B Prince HM Foss F Sokol L Greenwood M. Results from a pivotal, open-label, phase II study of romidepsin in relapsed or refractory peripheral T-cell lymphoma after prior systemic therapy. J Clin Oncol. (2012) 30:631–6. doi: 10.1200/JCO.2011.37.4223 22271479

[23] Bachy E Camus V Thieblemont C Sibon D Casasnovas RO Ysebaert L. Romidepsin plus CHOP versus CHOP in patients with previously untreated peripheral T-cell lymphoma: results of the ro-CHOP phase III study (Conducted by LYSA). J Clin Oncol. (2022) 40:242–51. doi: 10.1200/JCO.21.01815 34843406

[24] O'Connor OA Falchi L Lue JK Marchi E Kinahan C Sawas A. Oral 5-azacytidine and romidepsin exhibit marked activity in patients with PTCL: a multicenter phase 1 study. Blood. (2019) 134:1395–405. doi: 10.1182/blood.2019001285 31471376

[25] Marchion DC Bicaku E Turner JG Daud AI Sullivan DM Munster PN. Synergistic interaction between histone deacetylase and topoisomerase II inhibitors is mediated through topoisomerase IIbeta. Clin Cancer Res. (2005) 11:8467–75. doi: 10.1158/1078-0432.CCR-05-1073 16322310

[26] Cheriyath V Kuhns MA Kalaycio ME Borden EC. Potentiation of apoptosis by histone deacetylase inhibitors and doxorubicin combination: cytoplasmic cathepsin B as a mediator of apoptosis in multiple myeloma. Br J Cancer. (2011) 104:957–67. doi: 10.1038/bjc.2011.42 PMC3065279 21364585

[27] Dogan A Attygalle AD Kyriakou C. Angioimmunoblastic T-cell lymphoma. Br J Haematol. (2003) 121:681–91. doi: 10.1046/j.1365-2141.2003.04335.x 12780782

[28] Advani RH Skrypets T Civallero M Spinner MA Manni M Kim WS. Outcomes and prognostic factors in angioimmunoblastic T-cell lymphoma: final report from the international T-cell Project. Blood. (2021) 138:213–20. doi: 10.1182/blood.2020010387 PMC8493974 34292324

[29] Wudhikarn K Bunworasate U Julamanee J Lekhakula A Ekwattanakit S Khuhapinant A. Event free survival at 24 months is a strong surrogate prognostic endpoint of peripheral T cell lymphoma. Hematol Oncol. (2019) 37:578–85. doi: 10.1002/hon.2687 31702065

[30] Park SI Horwitz SM Foss FM Pinter-Brown LC Carson KR Rosen ST. The role of autologous stem cell transplantation in patients with nodal peripheral T-cell lymphomas in first complete remission: Report from COMPLETE, a prospective, multicenter cohort study. Cancer. (2019) 125:1507–17. doi: 10.1002/cncr.31861 PMC8269282 30694529

[31] El-Asmar J Reljic T Ayala E Hamadani M Nishihori T Kumar A. Efficacy of high-dose therapy and autologous hematopoietic cell transplantation in peripheral T cell lymphomas as front-line consolidation or in the relapsed/refractory setting: A systematic review/meta-analysis. Biol Blood Marrow Transplant. (2016) 22:802–14. doi: 10.1016/j.bbmt.2015.12.004 26713431

[32] Reimer P Rüdiger T Geissinger E Weissinger F Nerl C Schmitz N. Autologous stem-cell transplantation as first-line therapy in peripheral T-cell lymphomas: results of a prospective multicenter study. J Clin Oncol. (2009) 27:106–13. doi: 10.1200/JCO.2008.17.4870 19029417

